# *In Vitro* Activity and *In Vivo* Efficacy of Cefiderocol against Stenotrophomonas maltophilia

**DOI:** 10.1128/AAC.01436-20

**Published:** 2021-03-18

**Authors:** Rio Nakamura, Merime Oota, Shuhei Matsumoto, Takafumi Sato, Yoshinori Yamano

**Affiliations:** a Department of Anti-Infectious Drug Efficacy Evaluation Ι, Shionogi TechnoAdvance Research & Co., Ltd., Osaka, Japan; b Drug Discovery & Disease Research Laboratory, Shionogi & Co., Ltd., Osaka, Japan

**Keywords:** cefiderocol, *in vitro* activity, pharmacodynamics, *Stenotrophomonas maltophilia*, trimethoprim/sulfamethoxazole

## Abstract

Cefiderocol is a novel siderophore cephalosporin antibiotic with broad coverage against difficult-to-treat Gram-negative bacteria, including those resistant to carbapenems. Its activity against Stenotrophomonas maltophilia was investigated *in vitro* against clinical isolates and in lung infection models using strains either resistant (SR202006) or susceptible (SR201934, SR200614) to trimethoprim-sulfamethoxazole. Cefiderocol demonstrated potent *in vitro* activity against all 217 S. maltophilia clinical isolates tested (MIC_50_, 0.063 μg/ml; MIC_90_, 0.25 μg/ml). Cefiderocol also demonstrated low MICs against the trimethoprim-sulfamethoxazole-resistant S. maltophilia strains (i.e., SR202006; MIC, 0.125 μg/ml). In a neutropenic mouse lung infection model, cefiderocol (30 mg/kg body weight and 100 mg/kg) demonstrated a significant, dose-dependent reduction in the lung viable bacteria cell count compared with untreated controls in S. maltophilia infection and was the only antibiotic tested to show a similar significant effect in a trimethoprim-sulfamethoxazole-resistant S. maltophilia infection. In immunocompetent rat lung infection models of S. maltophilia, humanized dosing of cefiderocol (2 g every 8 h) and meropenem (1 g every 8 h) revealed pharmacokinetic profiles similar to those in human subjects, and the humanized cefiderocol dosing significantly reduced the lung viable bacteria cell count compared with baseline controls, which received no intervention. Together, the results from these studies suggest that cefiderocol could provide an effective alternative treatment option for S. maltophilia infections in the lower respiratory tract, particularly strains resistant to empirical antibiotics, such as trimethoprim-sulfamethoxazole or minocycline.

## Abstract (precis)

Cefiderocol is a novel siderophore cephalosporin antibiotic with broad coverage against difficult-to-treat Gram-negative bacteria, including those resistant to carbapenems. Its activity against Stenotrophomonas maltophilia was investigated *in vitro* against clinical isolates and in lung infection models using strains either resistant (SR202006) or susceptible (SR201934, SR200614) to trimethoprim-sulfamethoxazole.

## INTRODUCTION

Stenotrophomonas maltophilia is an opportunistic nonfermenter pathogen that causes a variety of different infections (1, 2). In hospitalized patients in the United States, the respiratory tract has been found to be the most frequent site of infection (3). The pathogen’s effects can be particularly problematic in patients who are immunosuppressed and those with indwelling medical devices (2). Many S. maltophilia infections are polymicrobial, with common copathogens, including other nonfermenting Gram-negative bacteria, such as Pseudomonas aeruginosa (4). Mortality rates in patients with S. maltophilia bacteremia vary widely but are in the region of 13 to 70% (5–7).

S. maltophilia displays resistance to many antibiotics (e.g., carbapenems) (1) by utilizing a number of intrinsic resistance mechanisms. These mechanisms include reduced membrane permeability, multidrug resistance efflux pumps, antibiotic-modifying enzymes, such as L1 and L2 β-lactamases, and the quinolone resistance gene *Smqnr* (1, 8). Recent data have suggested that mutations in the TonB membrane receptor of clinical S. maltophilia isolates are associated with a decrease in the uptake of ceftazidime and may also reduce susceptibility to siderophore antibiotics (9). While trimethoprim-sulfamethoxazole is active against it, S. maltophilia resistance to this antibiotic is growing and appears to be more common among isolates from inpatients than those from outpatients (5, 7). In addition, trimethoprim-sulfamethoxazole is associated with a variety of drug-drug interactions with ligands of CYP2C8, CYP2C9 enzymes or OCT2 transporter, including warfarin, methotrexate, diuretics, and several oral hypoglycemics, which may limit its use in some patients (10). Among patients with bacteremia, a higher Charlson comorbidity index and indwelling venous catheterization may predispose to infection by quinolone-resistant strains of S. maltophilia, which in turn may be associated with a significant risk of mortality (6). Thus, antibiotic treatment of respiratory, or other, infections caused by S. maltophilia may be compromised (11).

Cefiderocol is a novel, parenteral siderophore cephalosporin which is active against Gram-negative, nonfermenting pathogens, including S. maltophilia, even in the presence of carbapenemases due to its stability against these hydrolyzing enzymes (12). Cefiderocol has shown potent *in vitro* activity against a range of carbapenem-susceptible and carbapenem-resistant (CR) Gram-negative bacteria collected from around the world, including S. maltophilia strains resistant to other commonly used antibiotics (13–16). Preclinical studies have already demonstrated that cefiderocol is effective in murine thigh (17) and rat lung (18) infection models, and this *in vivo* efficacy appears to correspond well with its *in vitro* activity.

The objectives of the series of studies reported here were to characterize the *in vitro* activity of cefiderocol and its efficacy *in vivo* against various strains of S. maltophilia using animal models of lung infection, including a humanized dosing regimen in a rat model.

## RESULTS AND DISCUSSION

### *In vitro* activity of cefiderocol and comparator agents.

Cefiderocol exhibited *in vitro* activity in susceptibility tests against 217 S. maltophilia clinical isolates collected in 52 countries from patients with a variety of infections, with MIC_50_/MIC_90_ of 0.063/0.25 μg/ml. All S. maltophilia isolates were inhibited at a cefiderocol MIC of ≤2 μg/ml (range, 0.004 to 2.0 μg/ml). Activity against these isolates was also shown by trimethoprim-sulfamethoxazole (European Committee on Antimicrobial Susceptibility Testing [EUCAST]/Clinical and Laboratory Standards Institute [CLSI] susceptibility breakpoints 0.001/2 μg/ml, with resistance of ≥4 μg/ml) (MIC_50_ of 0.125/2.375 and MIC_90_ of 0.5/9.5 μg/ml), minocycline (CLSI susceptibility breakpoint 4 μg/ml) (MIC_50_/MIC_90_ of 0.25/1 μg/ml), and tigecycline (EUCAST pharmacokinetic [PK]/pharmacodynamic [PD] susceptibility breakpoint of 0.5 μg/ml) (MIC_50_/MIC_90_ of 1/2 μg/ml). All isolates were susceptible to minocycline (range, 0.063 to 4.0 μg/ml [CLSI breakpoint]), 99.1% to trimethoprim-sulfamethoxazole (range, ≤0.031/≤0.589 to 16/304 μg/ml [CLSI breakpoints]), and 73.9% to tigecycline (0.125 to 8.0 μg/ml [EUCAST PK/PD] breakpoint).

The results of activity testing with cefiderocol and other antibiotics against the S. maltophilia strains, which were used for *in vivo* studies, from patients with respiratory tract infections are shown in Table 1.

**TABLE 1:** MIC of experimental S. maltophilia strains isolated from patients with respiratory tract infections

Antibiotic	MIC (μg/ml) for:		
	S. maltophilia SR200614	S. maltophilia SR201934	S. maltophilia SR202006
Cefiderocol	0.063	0.5	0.125
Cefepime	32	64	32
Ceftazidime	128	128	64
Meropenem	64	>64	>32
Ciprofloxacin	2	2	32
Levofloxacin	2	1	16
Minocycline	0.125	0.25	2
Tigecycline	0.5	0.5	4
Trimethoprim-sulfamethoxazole	0.125/2.375	0.125/2.375	16/304
Colistin	>8	8	>32

### *In vivo* mouse lung infection model.

Two isolates were evaluated in a neutropenic mouse lung infection model, which showed different MIC profiles for some antibiotics which were active against S. maltophilia (Table 1). S. maltophilia strain SR202006 showed relatively high MIC values to trimethoprim-sulfamethoxazole, minocycline, levofloxacin, and ciprofloxacin within the MIC range of each antibiotic. In contrast, S. maltophilia strain SR201934 showed MIC values for each of these antibiotics corresponding to concentrations around the MIC_50_ values.

**FIG 1:**
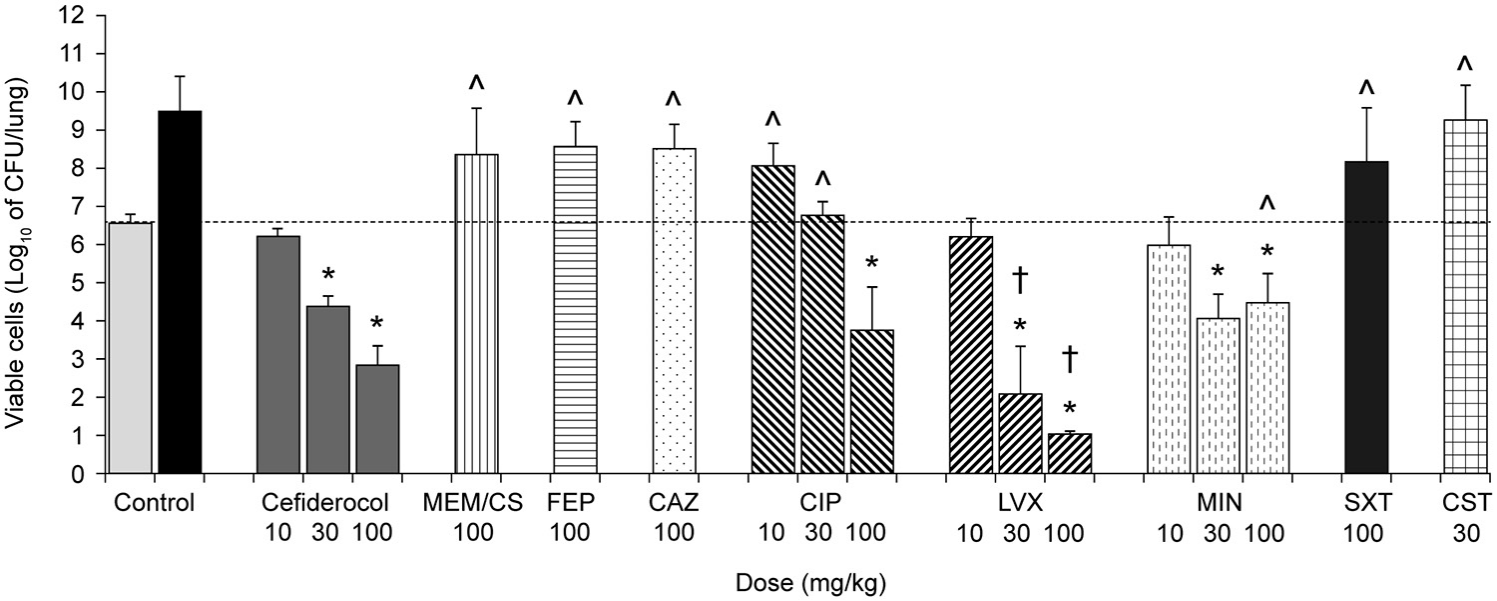
*In vivo* efficacy of cefiderocol in a neutropenic murine lung infection model caused by S. maltophilia strain SR201934. CAZ, ceftazidime; CIP, ciprofloxacin; CS, cilastatin; CST, colistin; FEP, cefepime; LVX, levofloxacin; MEM, meropenem; MIN, minocycline; SXT, trimethoprim-sulfamethoxazole. Data are expressed as the mean ± the standard deviation (SD). Five mice/group were tested. For controls, the number of viable bacterial cells in the lung was determined at 2 h after inoculation for untreated control mice (light gray bar) and at 24 h for vehicle-treated control mice (black bar). *, Significant reduction (*P < *0.05) versus untreated control (2 h), Dunnett’s multiple-comparison test; †, significant difference (*P < *0.05) versus the same dose of cefiderocol in favor of comparator, Welch’s *t* test; ^, significant difference (*P < *0.05) versus the same dose of cefiderocol in favor of cefiderocol, Welch’s *t* test.

**FIG 2:**
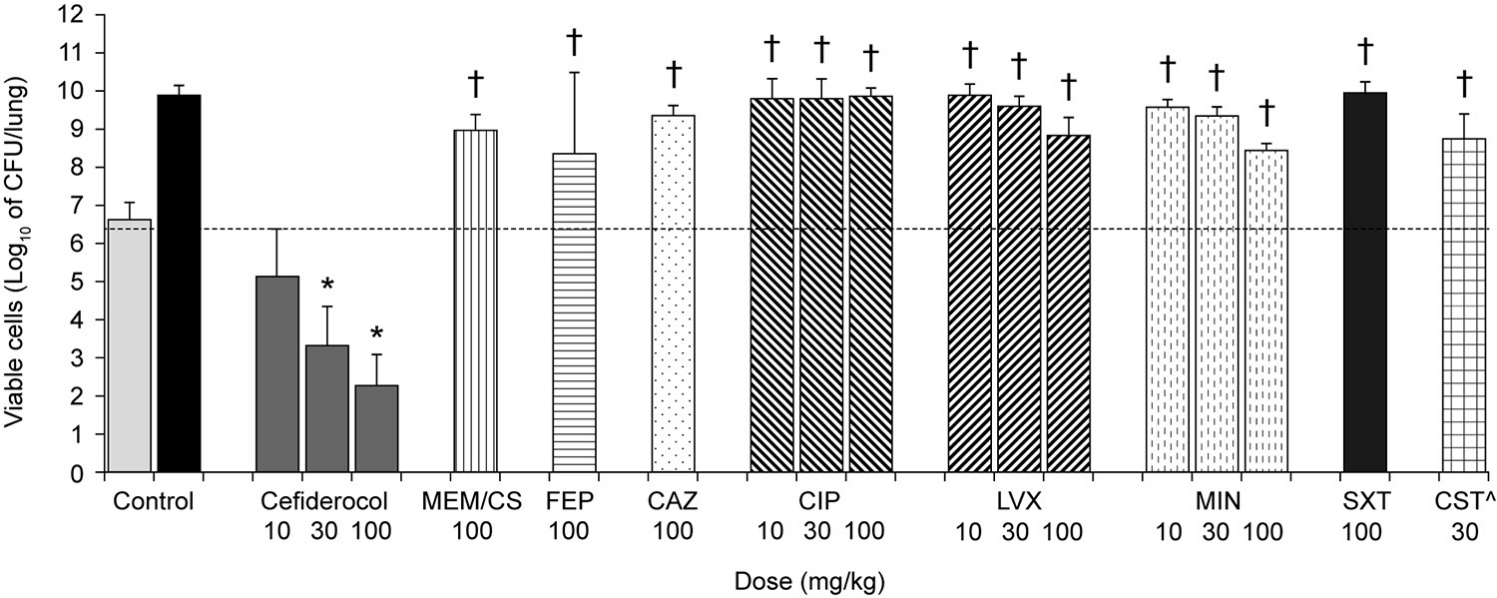
*In vivo* efficacy of cefiderocol in a neutropenic murine lung infection model caused by S. maltophilia strain SR202006. CAZ, ceftazidime; CIP, ciprofloxacin; CS, cilastatin; CST, colistin; FEP, cefepime; LVX, levofloxacin; MEM, meropenem; MIN, minocycline; SXT, trimethoprim-sulfamethoxazole. Data are expressed as the mean ± SD. Five mice/group were tested. For controls, the number of viable bacterial cells in the lung was determined at 2 h after inoculation for untreated control mice (light gray bar) and at 24 h for vehicle-treated control mice (black bar). *, Significant reduction (*P < *0.05) versus untreated control (2 h), Dunnett’s multiple-comparison test; †, significant difference (*P < *0.05) versus the same dose of cefiderocol, Welch’s *t* test. ^, *n* = 4 (the dose of 30 mg/kg showed toxicity in mice).

Against both isolates, administration of cefiderocol at 30 mg/kg and 100 mg/kg significantly reduced the viable bacterial cell count in the lung by 2- to 4-log_10_ CFU/lung compared with lungs of untreated control animals (Fig. 1 and 2). More potent efficacy by administration of minocycline, levofloxacin, and ciprofloxacin was observed for strain SR201934 than for strain SR202006, suggesting that the *in vivo* efficacy reflected well the *in vitro* activity of these compounds. Of note, efficacy was observed by ciprofloxacin administration for strain SR201934, although a susceptibility breakpoint for this antibiotic has not been defined for S. maltophilia. On the other hand, meropenem, cefepime, ceftazidime, and colistin did not show efficacy against these strains (Fig. 1 and 2), which was likely due to the high MICs (Table 1). The finding that trimethoprim-sulfamethoxazole was not effective *in vivo*, despite demonstrating a low MIC against S. maltophilia SR201934 *in vitro*, may be because infected mice can display increases in the concentration of thymidine (19), high levels of which have been shown to have an antagonistic impact on the activity of trimethoprim-sulfamethoxazole *in vitro* (20).

### *In vivo* immunocompetent rat lung infection model.

**FIG 3:**
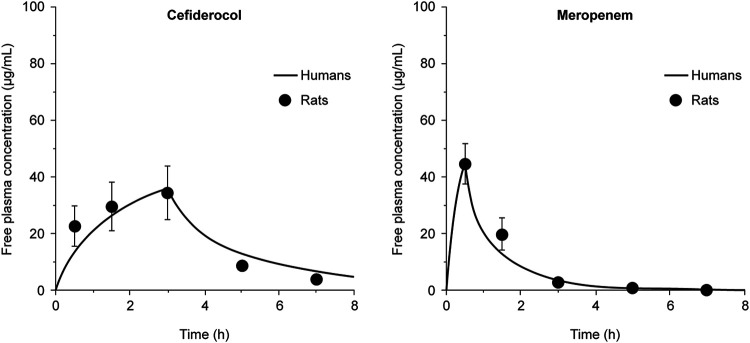
Pharmacokinetic profiles of humanized doses of cefiderocol at 2 g every 8 h (simulated 3-h infusion) and meropenem 1 g every 8 h (0.5-h infusion) recreated in immunocompetent rats. Data points are expressed as the mean ± SD of three readings/rat. Data for cefiderocol were adapted from (18).

**FIG 4:**
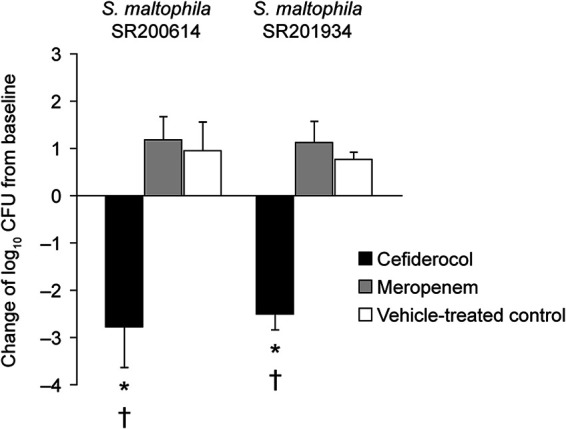
*In vivo* efficacy of cefiderocol and meropenem, recreating human plasma pharmacokinetics in an immunocompetent rat respiratory-infection model caused by the S. maltophilia strains SR200614 and SR201934. Data are expressed as the mean ± SD. Three to six rats/group were tested. The number of viable cells in lungs was determined 96 h after infection. *, Significant reduction (*P < *0.05) versus baseline control value, Welch’s *t* test; †, significant difference (*P < *0.05) versus meropenem, Welch’s *t* test.

Humanized doses of cefiderocol and meropenem (both dissolved in 0.9% saline) were tested against two strains of trimethoprim-sulfamethoxazole-susceptible S. maltophilia (SR200614 and SR201934) in the rat lung infection model (Table 1). The plasma concentration-time profiles between the simulated 3-h infusion of cefiderocol (2 g every 8 h) and the simulated 0.5-h infusion of meropenem (1 g every 8 h) were similar to human PK profiles (Fig. 3). In this rat model, the lungs of untreated animals showed hemorrhagic inflammation and a 1-log_10_ CFU/lung increase in bacterial numbers over 96 h. Against both strains of S. maltophilia, humanized cefiderocol dosing reduced the viable cell count by 2- to 3-log_10_ CFU/lung compared with baseline controls, which received no intervention (Fig. 4). This suggests that cefiderocol administered according to the approved dosing regimen has the clinical potential to effectively treat respiratory tract infections caused by S. maltophilia. As expected from the MIC values (Table 1), meropenem showed no significant activity compared with controls.

We previously demonstrated the bactericidal activity of cefiderocol *in vivo* against a range of Gram-negative bacteria in murine thigh and rat lung infection models (17, 18, 21, 22), including CR strains of *Klebsiella* spp., Pseudomonas aeruginosa, and Acinetobacter baumannii. In these previous investigations, the *in vivo* efficacy of cefiderocol corresponded well with the *in vitro* activity measured by using iron-depleted cation-adjusted Muller-Hinton broth based on CLSI recommendations, an observation confirmed by the current series of experiments against S. maltophilia strains, which were either susceptible or resistant to trimethoprim-sulfamethoxazole.

The cefiderocol dosing regimen (100 mg/kg) selected for the murine lung infection model has been shown to achieve approximately 54% of the cumulative percentage of a 24-h period that the free drug concentration in plasma exceeds the MIC (*fT*>MIC) against four strains of S. maltophilia, with cefiderocol MIC values of 0.125 and 0.25 μg/ml (17). In the previously described rat model, the percentage *fT*>MIC for cefiderocol at 2 g once every 8 h infused over 3 h was 100% for A. baumannii and P. aeruginosa strains with MICs of ≤4 μg/ml (18). In a neutropenic murine thigh infection model, infected by S. maltophilia strains, cefiderocol demonstrated bactericidal efficacy *in vivo* and reduced the bacterial burden against 87.5% of the tested strains (22).

In summary, the excellent *in vitro* activity of cefiderocol against a global collection of S. maltophilia isolates in surveillance studies (14) was shown to extend to trimethoprim-sulfamethoxazole-resistant strains, and the *in vivo* efficacy of cefiderocol in murine and rat models was associated with its *in vitro* activity against these isolates. Other β-lactam agents were inactive against the S. maltophilia isolates, and concentration-dependent effects were demonstrated with levofloxacin, ciprofloxacin, and minocycline. The findings indicate that cefiderocol could provide an effective alternative treatment option for S. maltophilia infections in the lower respiratory tract, particularly against strains resistant to empirical antibiotics such as trimethoprim-sulfamethoxazole or minocycline. However, evidence from clinical studies is required to confirm its place in clinical practice for the management of patients with respiratory tract infections.

## MATERIALS AND METHODS

### Agents.

The following antimicrobial agents were tested: cefiderocol, cefepime, ceftazidime, ciprofloxacin, colistin, meropenem, minocycline, tigecycline, levofloxacin, and trimethoprim-sulfamethoxazole. In *in vivo* studies, meropenem was administered with cilastatin at the same dosage to minimize meropenem degradation by murine dihydropeptidase-1 (DHP-1). Cefiderocol was synthetized by Shionogi & Co., Ltd. (Osaka, Japan) for both *in vitro* and *in vivo* studies. For the *in vitro* and/or *in vivo* studies, cefepime, ceftazidime, ciprofloxacin, colistin, meropenem, cilastatin, minocycline, tigecycline, and levofloxacin were obtained from commercial sources, and trimethoprim-sulfamethoxazole was synthesized by Shionogi & Co., Ltd. For the *in vivo* studies, ceftazidime was obtained from GlaxoSmithKline K.K. (London, UK), meropenem from Dainippon Sumitomo Pharma Co., Ltd. (Osaka, Japan), minocycline from Pfizer, Inc. (New York, USA), and trimethoprim-sulfamethoxazole from Taiyo Pharma Co., Ltd. (Tokyo, Japan).

### S. maltophilia test strains.

All three of the S. maltophilia strains were collected from patients with respiratory tract infections in the SIDERO-CR surveillance study (14). Strain SR202006 (South Africa) was trimethoprim-sulfamethoxazole resistant, and SR201934 (Japan) and SR200614 (United States) were trimethoprim-sulfamethoxazole susceptible.

### *In vitro* studies.

Full details of the susceptibility testing methodology were published previously (13, 14). It was complemented by testing with tigecycline, minocycline, and trimethoprim-sulfamethoxazole at Shionogi & Co. (Osaka, Japan) using CLSI standard methods. Cation-adjusted Mueller-Hinton broth (CAMHB) (Becton, Dickinson, Sparks, MD, USA) was used for all antimicrobial susceptibility testing. For cefiderocol, iron-depleted CAMHB (ID-CAMHB) (at a final iron concentration of ≤0.03 μg/ml) was used and was prepared according to the CLSI Subcommittee on Antimicrobial Susceptibility Testing-approved methodology (14).

### *In vivo* studies.

Full details of the methodology for the neutropenic murine lung infection model (17) and the immunocompetent rat lung infection model (18) were published previously. All studies with animals were approved by the Institutional Animal Care and Use Committee of Shionogi & Co., Ltd. The S. maltophilia strains used in the *in vivo* studies were subjected to multilocus sequence typing (MLST) using the PubMLST typing database (https://pubmlst.org/). Direct sequencing of PCR products was performed by Eurofins Genomics (Tokyo, Japan). SR201934 was characterized as belonging to the S. maltophilia *sensu stricto* complex (Sm6). The strain sequence types (ST) were confirmed by sequencing seven housekeeping genes (*atpD*, *gapA*, *guaA*, *mutM*, *nuoD*, *ppsA*, and *recA* of S. maltophilia). For two strains, SR200614 and SR202006, it was not possible to confirm the sequence typing number; the fact that all three strains were human-derived and were found to harbor L2 beta-lactamase genes, strongly suggests that all were from the S. maltophilia *sensu lato* lineage (23).

### Neutropenic murine lung infection model.

Briefly, for the neutropenic murine lung infection model, male Jcl:ICR mice (weighing 17 to 20 g [CLEA Japan, Inc., Tokyo, Japan]) were rendered neutropenic by two doses of intraperitoneal cyclophosphamide prior to the experiment (150 mg/kg on day −4 and 100 mg/kg on day −1). Anesthetized mice were inoculated with 3 to 5 × 10^6^ CFU of S. maltophilia. Antibiotics (in a 0.9% saline vehicle) were administered subcutaneously at 2, 5, and 8 h postinfection (5 animals/dosing group). Control mice were not treated with antibiotics and received either no intervention (untreated) or vehicle (vehicle-treated). Following the initial infection, mice were euthanized at 2 h (untreated controls) or 24 h (antibiotic-treated animals or vehicle-treated controls), lungs were excised, and the numbers of viable cells in lung tissue were counted. Dunnett’s multiple-comparison test was used to test differences between active treatment and untreated baseline control groups in the number of viable cells following treatment. Welch’s *t* test was used to compare differences between comparator antibiotics cefiderocol at the same doses. The *P* value for significance in both tests was <0.05.

### Immunocompetent rat lung infection model.

Specific-pathogen-free, 5-week-old, male Sprague-Dawley rats (weighing approximately 150 g; Charles River Laboratories Japan, Inc., Kanagawa, Japan) were anesthetized before being infected via an intratracheal route with 0.1 ml of 3 × 10^7^ CFU/ml inoculum with molten nutrient agar. Cefiderocol or meropenem was administered via an inferior jugular vein cannula implanted 3 to 6 days prior to the initiation of treatment, according to schedules designed to mimic their PK profiles at the approved doses in healthy human subjects: 2 g cefiderocol every 8 h as a 3-h infusion and 1 g meropenem every 8 h as a 0.5-h infusion (18). The PK profiles were adjusted according to the protein binding ratios in humans and rats taken from the literature (18, 24), and the resulting profiles represent free-drug plasma concentrations. Antibiotic treatment was initiated 2 h postinfection and was continued for 96 h for the recreated human PK profile (3 to 6 rats/dosing group). In a control group, infected animals received saline vehicle at a constant flow rate of 0.4 ml/hour (3 to 6 rats/dosing group). At 96 h after treatment initiation, rats were anesthetized and, after exsanguination, lungs were collected and the numbers of viable cells in lung tissue were counted. Five additional infected rats had their organs harvested at the initiation of dosing without any treatment and were used as baseline controls. Welch’s *t* test was used to compare differences in bacterial density at 96 h between the treatment and baseline controls, and between the cefiderocol and meropenem treatment groups. The *P* value for significance was <0.05.

### Measurement of cefiderocol and meropenem concentrations in the rat experimental model recreating human pharmacokinetics.

The cefiderocol concentration in rat plasma was described elsewhere (18). In brief, rats were treated with cefiderocol, and on the second day of dosing, blood samples were collected at various times after the start of the infusion. Cefiderocol concentrations in plasma were determined by the validated liquid chromatography-tandem mass spectrometry (LC-MS-MS) method.

For measurement of meropenem plasma concentrations, rats were treated with meropenem as described above, and blood samples were collected and centrifuged. This was followed by the collection of a 10-μl aliquot of each plasma sample, which was frozen immediately on dry ice and stored at −20°C prior to analysis. Concentrations of the drug in plasma were determined by the LC-MS-MS method. Samples were deproteinated with acetonitrile-0.1% formic acid (vol/vol). For plasma calibration, appropriate concentrations of meropenem were spiked into rat plasma to give eight standards from 0.1 to 300 μg/ml. Quality control (QC) samples were prepared by spiking rat plasma with meropenem to achieve final concentrations of 0.3 (low QC), 10 (medium QC), and 300 (high QC) μg/ml. The LC-MS-MS system comprised a Shimadzu Corporation high-pressure liquid chromatography (HPLC) system (LC-20A) in tandem with an API 5000 triple-quadrupole MS in electrospray ionization mode. Chromatographic separation was performed using an Acquity C_18_ column (inside diameter, 2.1 mm; length, 50 mm; particle size, 1.7 μm) with a gradient using mobile phases of 0.1% formic acid and acetonitrile-0.1% formic acid at a flow rate of 0.4 ml/min. Meropenem concentrations were obtained using LC-MS-MS monitoring the product ion transitions of *m/z* 384.2 and *m/z* 141. The analysis run time was 2.4 min.

### Data availability.

All data obtained in these experiments are included in the manuscript.
